# Working Family Caregiver Well-Being: Work Impact, Workplace Supports, and Family Discord

**DOI:** 10.1093/geroni/igab046.1384

**Published:** 2021-12-17

**Authors:** Jiayun Xu, Pi-Ju Liu, Yisheng Peng, Scott Beach

**Affiliations:** 1 Purdue University, West Lafayette, Indiana, United States; 2 George Washington University, Washington, District of Columbia, United States; 3 University of Pittsburgh, University of Pittsburgh, Pennsylvania, United States

## Abstract

A recent study by our team showed that family discord about care provision among multiple family caregivers was a significant predictor of caregiver burden, anxiety, depression, and activity restrictions. This study examined the combined effects of family discord, work impacts (missed work, caregiving affects work), and workplace supports (flexible hours, caregiver benefits) on caregiver well-being. We conducted a secondary analysis using cross-sectional survey data from the Western Pennsylvania Family Caregiving Project 2017-2018. Participants were family caregivers who worked outside the home and shared older adult (i.e. over age 50) caregiving responsibilities with family (n=364, mean age: 52.59 years, female: 71.7%, White: 79.7%, cared for a parent: 69.5%, mean work hours/week: 37.30). Hierarchical regression analyses were conducted testing for main effects of family discord, work impacts, and workplace supports; and interactions between discord and work impacts/workplace supports. The presence of family discord negatively impacted all caregiver well-being outcomes (p<0.05). Having more work impacts increased the risk for anxiety (p<0.04), activity restrictions (p<0.01) and burden (p<0.01). No main effects were found for workplace support. Moderating effects were found, such that anxiety was high when family discord and work impacts were higher (p=0.025). Additionally, more activity restrictions occurred when caregivers had low workplace support and higher family discord (p=0.020). Results suggest having less family discord, more workplace support, and less negative work impacts may improve caregiver well-being. Future work is needed to determine which work supports are most beneficial to this population and how family discord and negative work impacts can be reduced.

